# Molecular screening for *Wolbachia* endosymbionts in phlebotomine sand flies within the North Central Province, Sri Lanka

**DOI:** 10.3389/frmbi.2026.1905698

**Published:** 2026-10-01

**Authors:** F. B. R. P. Kumara, D. K. Hettiarachchi, N. D. A. D. Wijegunawardana

**Affiliations:** 1 Department of Bioprocess Technology, Faculty of Technology, Rajarata University of Sri Lanka, Mihintale, Sri Lanka; 2 Department of Biological Sciences, Faculty of Applied Sciences, Rajarata University of Sri Lanka, Mihintale, Sri Lanka

**Keywords:** 16S rRNA gene amplicon sequencing, leishmaniasis, *Phlebotomus*, sandflies, *Serratia*, Sri Lanka, wsp *Wolbachia*

## Abstract

Cutaneous leishmaniasis is a growing public health threat in Sri Lanka, transmitted primarily by phlebotomine sand flies. While biological control using *Wolbachia* has proven effective against other vector-borne diseases, its potential application in phlebotomine sand flies remains largely unexplored. Therefore, investigating the natural prevalence of these endosymbionts in Sri Lankan sand fly populations is well justified. To address this knowledge gap and evaluate the feasibility of symbiont-mediated control, we conducted a six-month entomological survey across four endemic sampling areas in the North Central Province. Captured sand flies were morphologically identified at genus level, and 90 females belonging to the genera *Phlebotomus* and *Sergentomyia* were screened for *Wolbachia* using *wsp*-targeted polymerase chain reaction (PCR). To provide complementary evidence and characterize the broader sand fly-associated bacterial community, high-throughput 16S rRNA gene amplicon sequencing was performed on a pooled sample of 25 females. *Wolbachia* was not detected in any of the individually screened specimens (0/90; observed prevalence, 0.0%; approximate rule-of-three upper 95% prevalence limit, 3.3%) or in the pooled 16S dataset. Sequencing generated 220,698 non-chimeric reads, and *Serratia* was the most abundant identifiable genus, accounting for 41.71% of bacterial reads. These findings indicate an absence of detectable *Wolbachia* under the methods applied, rather than its definitive absence from the wider regional sand fly population. The study provides baseline evidence relevant to evaluating the feasibility and limitations of symbiont-mediated strategies for leishmaniasis vector control in Sri Lanka.

## 1 Introduction

Leishmaniasis is a major vector-borne disease that threatens more than one billion individuals in over 100 endemic nations. There are three types of leishmaniasis: visceral leishmaniasis (VL), which is the most severe; cutaneous leishmaniasis (CL), which is the most common; and mucocutaneous leishmaniasis (MCL), which causes severe tissue damage. Every year, there are approximately 50,000 to 90,000 new cases of VL and 700,000 to 1.2 million new cases of CL worldwide. The most affected areas are South Asia, East Africa, the Mediterranean basin, and parts of South America (Georgiadou et al., 2015; Cosma et al., 2024; Gunathilaka et al., 2025).

Since the first reported case in 1992, CL has emerged as a major parasitic public health concern in Sri Lanka. Unlike the visceral-dominant pattern observed in neighboring India, the disease in Sri Lanka is predominantly cutaneous, caused by a unique epidemiological variant of *Leishmania donovani* (Gunathilaka et al., 2025). The incidence of the disease has surged dramatically; reported cases increased 15-fold between 2001 and 2010, and a further 10-fold from 2010 to 2019, with the most significant outbreaks occurring during the 2017–2018 period. Current epidemiological surveillance indicates an annual incidence exceeding 4,000 new CL cases (Karunaweera et al., 2021).

Transmission involves interactions among *Leishmania* parasites, phlebotomine sand fly vectors, and human hosts. In Sri Lanka, *Phlebotomus argentipes* is recognized as the principal vector of leishmaniasis. The molecular detection of *L. donovani* DNA in *P. argentipes* supports its involvement in Sri Lanka’s transmission cycle (Wijerathna et al., 2021). However, whether transmission in Sri Lanka is predominantly anthroponotic, zoonotic, or maintained through a combination of both cycles remains unresolved (Amarasinghe and Wickramasinghe, 2020; Ganehiarachchi, 2021).

The North Central Province (encompassing the Anuradhapura and Polonnaruwa districts) serves as a major hotspot for leishmaniasis transmission. The region’s dry tropical climate and seasonal fluctuations significantly influence the ecology of phlebotomine sandflies and the overall epidemiology of leishmaniasis. Climatic variables, such as temperature, relative humidity and rainfall influence vector population dynamics and disease risk. Furthermore, extensive agricultural environments including chena farming, coconut plantations, and widespread irrigation networks, create suitable breeding and resting microhabitats for these vectors (Ranawakal et al., 2007; Karunaweera et al., 2020).

Traditional methods of control, such as spraying insecticides and using bed nets, have limitations due to hidden breeding sites, insecticide resistance, and the widespread distribution of vectors (Kishore et al., 2006). As a result, biological control methods, particularly those utilizing *Wolbachia* endosymbionts, which have been successfully implemented against *Aedes* mosquitoes, have gained popularity. *Wolbachia*, an obligate intracellular bacterium, infects 40–76% of arthropods, making it a widespread symbiont (Wong et al., 2023).

*Wolbachia* manipulates host reproduction through distinct mechanisms, including cytoplasmic incompatibility (CI), male killing, feminization, and the induction of parthenogenesis, thereby enhancing its maternal transmission within host populations. These reproductive alterations confer a reproductive advantage on infected females and facilitate the maternal transmission and spread of *Wolbachia* within host populations (Werren et al., 2008; Brelsfoard and Dobson, 2009). CI, the most common reproductive manipulation induced by *Wolbachia*, results in embryonic mortality or reduced egg hatch when infected males mate with uninfected females or females harboring incompatible *Wolbachia* strains (Chen et al., 2019).

Reproductive manipulation, particularly cytoplasmic incompatibility, facilitates the spread of *Wolbachia* through host populations by providing infected females with a reproductive advantage (Walker et al., 2011). Furthermore, certain *Wolbachia* strains inhibit the replication or transmission of selected pathogens, particularly viruses and protozoan parasites, through mechanisms that may include competition for host cellular resources and modulation of innate immune responses (Lindsey et al., 2018).

Research on the prevalence and genetic diversity of *Wolbachia* in phlebotomine sand flies remains geographically and taxonomically limited. Nevertheless, available molecular surveys have revealed marked variation in infection prevalence among sand fly species and geographical locations (Ono et al., 2001; Torres-Llamas et al., 2025). PCR-based studies conducted in Iran, Türkiye, Spain, and Morocco have reported prevalence estimates ranging from low or undetectable levels in some populations to approximately 70–81% in others, with detected strains belonging predominantly to *Wolbachia* supergroups A and B (Bordbar et al., 2014; Karatepe et al., 2018; Torres-Llamas et al., 2025). In addition to targeted *Wolbachia*-specific PCR assays, 16S rRNA amplicon sequencing can provide complementary evidence by detecting *Wolbachia* within the broader sand fly-associated bacterial community (Maleki-Ravasan et al., 2024). Sequence-based analysis of sand flies from Spain and Morocco identified five wsp haplotypes, while the occurrence of closely related haplotypes across different host species was considered consistent with possible horizontal transmission (Torres-Llamas et al., 2025).

The application of *Wolbachia*-based biocontrol against leishmaniasis presents distinct biological challenges because Leishmania develops predominantly as an extracellular parasite within the sand fly alimentary tract, whereas many well-characterized *Wolbachia*-mediated pathogen-blocking interactions involve intracellular pathogens and competition for host cellular resources (Lindsey et al., 2018; Tom et al., 2023). Nevertheless, experimental transinfection of *Lutzomyia longipalpis*-derived cell lines with the *wMel*Pop-CLA strain successfully established stable *Wolbachia* infections and altered the expression of genes associated with the IMD, Toll, and Jak–STAT innate immune pathways (Da Silva Gonçalves et al., 2019). However, these immune alterations did not significantly reduce Leishmania infantum adhesion or parasite load under the tested *in vitro* conditions, indicating that the consequences of *Wolbachia* infection for Leishmania development and transmission require further investigation in whole sand flies (Da Silva Gonçalves et al., 2019).

To our knowledge, no previous studies have evaluated the natural prevalence of *Wolbachia* in Sri Lankan sand fly populations, leaving a critical knowledge gap regarding the feasibility of symbiont-mediated biocontrol strategies in this region. Therefore, this study aimed to investigate the prevalence of *Wolbachia* in sand flies and fly populations across the selected areas of highly endemic North Central Province. To ensure the robust verification of our molecular findings, standard polymerase chain reaction (PCR) screenings were supplemented with 16S rRNA gene amplicon sequencing on a pooled sand fly sample. This dual methodological approach was employed to assess the detectability of *Wolbachia* sequences while concurrently profiling the broader bacterial community associated with the pooled sample.

## 2 Materials and methods

### 2.1 Ethical clearance for the study

Ethical approval for this research project was granted by the Ethics Review Committee of the Institute of Biology Sri Lanka (ERC-IOBSL) under registration number: ERC/IOBSL/441/07/2025. All research activities were conducted in strict accordance with the guidelines and conditions stipulated by the committee, including completion of the study within the approved timeframe and the requirement to obtain prior authorization from the IOBSL for any modifications to the approved research protocol.

### 2.2 Sand fly collection

Sample collection was conducted across four Public Health Inspector (PHI) areas in Sri Lanka’s North Central Province Medawachchiya (8°33’59.7”N, 80°29’02.6”E), Mihintale (8°23’24.6”N, 80°31’11.1”E), Habarana (8°01’22.5”N, 80°47’20.5”E), and Bakamuna (7°48’43.7”N, 80°50’35.5”E) (**Figure 1**). The North Central Province is an established CL-endemic region where ecological, climatic, and land-use factors may support sand fly persistence and disease transmission (Ganehiarachchi, 2021; Nayakarathna et al., 2022). The region is characterized by agricultural landscapes, irrigation systems, scrub vegetation, wet-soil habitats, leaf litter, and termite mounds, several of which have been identified as potential resting or breeding habitats for phlebotomine sand flies in Sri Lanka (Galgamuwa et al., 2018; Wijerathna and Gunathilaka, 2020a), The proximity of these vector-supporting habitats to agricultural and other outdoor human activities may increase opportunities for human–sand fly contact (Galgamuwa et al., 2018; Wijerathna et al., 2020). Consequently, these sites were considered suitable sentinel locations for investigating sand fly ecology and the occurrence of natural *Wolbachia* infections.

**Figure 1:**
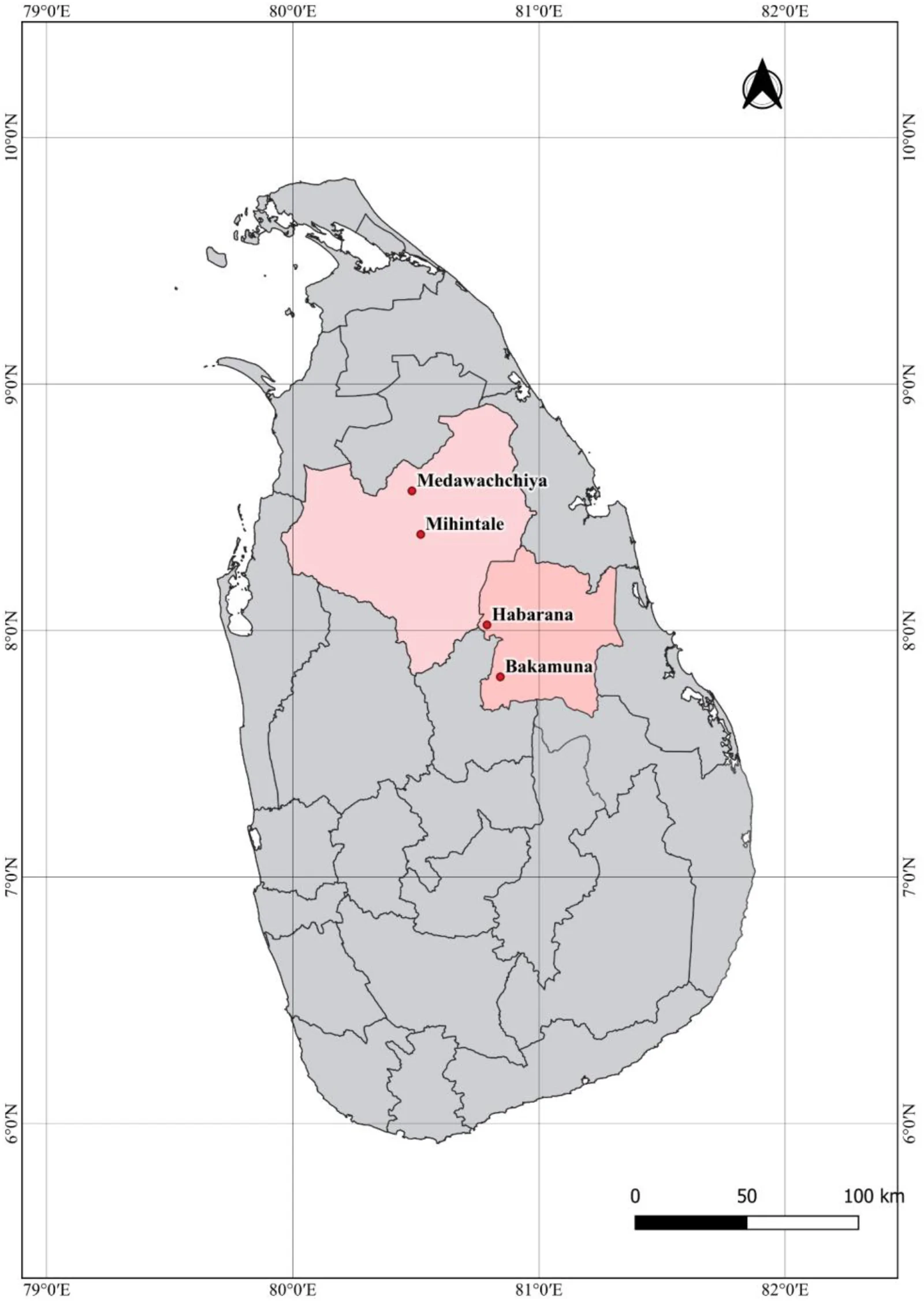
Map showing the locations of sand fly collection sites in the North Central Province, Sri Lanka, where specimens were sampled for Wolbachia screening.

Sand fly surveillance was conducted monthly using light traps, Following established collection protocols for phlebotomine vectors (Alten et al., 2015). During each sampling event, two CDC light traps were deployed per site and positioned approximately 50 m apart in optimal microhabitats, targeting areas adjacent to potential breeding grounds, human dwellings, and domestic animal shelters. To align with the nocturnal activity patterns of adult sand flies, the traps were operated continuously over a 12 h overnight period (Aklilu et al., 2017). Following collection, the captured samples were securely transported to the laboratory using temporary containers. Specimens were cataloged using unique identifiers and promptly stored in PBS at -20 °C to preserve both structural and molecular integrity before identification.

### 2.3 Morphological identification

Prior to morphological examination, collected phlebotomine sand fly specimens were rinsed carefully with alcohol to remove residual debris and facilitate clear visualization of fine taxonomic features. Morphological identification was conducted up to genus level following the taxonomic keys developed by Wijerathna and Gunathilaka (2020b), Diagnostic categorization was primarily based on the morphological characteristics of wing venation in female sand flies and external genital structures in male sand flies All examinations were performed using a Nikon stereomicroscope (Nikon Corporation, Tokyo, Japan) at the Department of Bioprocess Technology, Faculty of Technology, Rajarata University of Sri Lanka. Species-level identification was not performed; therefore, the specimens were reported only as belonging to the genera.

### 2.4 DNA extraction

Genomic DNA was extracted from individual female sand fly specimens (n=90) to enable specimen-specific *Wolbachia* screening. Each individual insect was mechanically homogenized in 30 μL of GT buffer supplied with the Presto™ Mini gDNA Bacterial Kit (Geneaid Biotech Ltd., Taiwan). Subsequent extraction steps were performed in accordance with the manufacturer’s guidelines. The purified genomic DNA was ultimately eluted in 50 μL of the provided elution buffer. To ensure the suitability of the extracted DNA for downstream molecular analyses, the concentration and purity of the eluted samples were quantitatively assessed prior to PCR amplification using a Jenway Genova Nano micro-volume spectrophotometer (Cole-Parmer Ltd., UK).

### 2.5 PCR amplification and *Wolbachia* detection

PCR screening for *Wolbachia* was performed by targeting a fragment of the *Wolbachia* surface protein gene (*wsp*), a commonly used molecular marker for the detection and preliminary characterization of *Wolbachia* strains. Amplification was performed using a Heal Force T960 thermal cycler (Heal Force Bio-meditech Holdings, China) with the *wsp*-specific primers 81F (5′-TGG TCC AAT AAG TGA TGA AGA AAC-3′) and 691R (5′-AAA AAT TAA ACG CTA CTC CA-3′). The expected amplicon size ranged from 590 to 632 bp (Zhou and Neill, 1998).

Each 25-µL PCR mixture contained 12.5 µL of 2× GoTaq^®^ Green Master Mix (Promega, Madison, WI, USA), 0.5 µL of each primer from a 10-µM working stock, corresponding to a final concentration of 0.2 µM per primer, 2 µL of genomic DNA template, and 9.5 µL of nuclease-free water. Thermal cycling consisted of an initial denaturation at 95 °C for 2 min, followed by 38 cycles of denaturation at 95 °C for 30 s, annealing at 54 °C for 45 s, and extension at 72 °C for 1 min, with a final extension at 72 °C for 10 min.

Genomic DNA extracted using dissected ovaries of three *Wolbachia*-infected female *Culex pipiens* specimens was included as the positive control, whereas nuclease-free water was included as the no-template negative control in each PCR run. Amplified products were separated on a 1.5% agarose gel prepared in 1× TBE buffer at 90 V for 30 min. A 100-bp DNA ladder was used to estimate amplicon size. DNA bands were visualized under 302-nm UV illumination using a Labnet ENDURO™ GDS2 Gel Documentation System (Labnet International, Edison, NJ, USA) with 6x GeneDireX Novel Juice.

### 2.6 pooled 16S rRNA gene amplicon sequencing

Following negative results from individual PCR assays targeting the *wsp* gene, 25 female sand flies were pooled prior to DNA extraction for complementary 16S rRNA gene amplicon sequencing and characterization of the sand fly-associated bacterial community. Due to the diminutive anatomical size of phlebotomine sand flies, the absolute microbial biomass and the corresponding bacterial genomic DNA yield from a single individual are exceptionally low, frequently falling below the requisite minimum input thresholds for robust high-throughput 16S rRNA library preparation. Therefore, a pooled sample consisting of freshly collected female *Phlebotomus* spp. individuals (n=25) was used for DNA extraction (Barb et al., 2016). Genomic DNA was extracted from the pooled sample following the previously described mechanical homogenization and column-based extraction protocol.

Library preparation for 16S rRNA gene amplicon sequencing was performed utilizing the Ion 16S™ Metagenomics Kit and the Ion Plus™ Fragment Library Kit (Thermo Fisher Scientific, Inc., MA, USA). This methodology targets multiple hypervariable regions (V2, V3, V4, V6-V7, V8, and V9) of the bacterial 16S rRNA gene to provide broad coverage of bacterial diversity. Following successful library construction, Sequencing was executed using an Ion GeneStudio™ S5 system (Thermo Fisher Scientific, USA).

### 2.7 Bioinformatics and 16S rRNA gene amplicon sequencing data analysis

Single-end reads were analyzed using the QIIME 2 amplicon distribution version 2024.10 (q2cli version 2024.10.1). The sequencing provider confirmed that sequencing adapters had been removed by the default Torrent Suite workflow. Raw read lengths ranged from 25 to 330 nucleotides (median, 210 nucleotides), reflecting the mixed hypervariable-region design of the Ion 16S Metagenomics Kit. Fixed 3′ truncation was not applied because truncation at 200 nucleotides would have retained only 60.16% of the reads, whereas truncation at 250 nucleotides would have retained only 11.38%. Based on sensitivity analysis, a uniform 20-nucleotide 5′ trim was applied in the primary analysis; this was used as a general preprocessing step and was not interpreted as primer-specific excision. A parallel untrimmed analysis was retained as a sensitivity control.

Reads were denoised using the QIIME 2 DADA2 denoise-pyro method with trunc-len = 0, trim-left = 20, max-ee = 2, trunc-q = 2, max-len = 0, independent pooling, and consensus-based chimera removal. Taxonomy was assigned using a Naive Bayes classifier trained against the SILVA 138 reference database clustered at 99% sequence identity, with a confidence threshold of 0.7 and automatic read-orientation detection. For bacterial community summaries, only features assigned to the domain Bacteria were retained, and mitochondrial and chloroplast assignments were excluded. Relative abundances were calculated from the resulting non-organelle bacterial feature table. Formal alpha- and beta-diversity comparisons were not undertaken because the dataset consisted of a single pooled sample.

### 2.8 Statistical analysis

Data were analyzed using IBM SPSS Statistics version 32.0. Sand fly abundance, genus composition, and sex distribution were summarized as frequencies and percentages. Differences in monthly sand fly abundance among the four study sites were assessed using the Friedman test, with sampling month treated as the blocking factor. The association between genus and sex was examined using Pearson’s chi-square test, excluding two damaged specimens that could not be identified to genus. Cramer’s V was calculated to quantify the strength of this association. Statistical significance was set at *p* < 0.05.

## 3 Results

### 3.1 Sample collection

A total of 331 sand flies were collected from Medawachchiya, Mihintale, Habarana, and Bakamuna between October 2024 and March 2025. Sand fly abundance varied across sampling months, with the highest monthly catch recorded in January 2025 (n = 99; 29.9%) and the lowest in November 2024 (n = 6; 1.8%). Although Habarana yielded the highest cumulative catch and the largest catch recorded during a single site–month sampling occasion (n = 43), monthly sand fly abundance did not differ significantly among the four sampling areas (Friedman test: χ² (3) = 1.17, p = 0.761, six monthly sampling occasions) (**Figure 2**). The overall collection was strongly male-biased, comprising 241 males (72.8%) and 90 females (27.2%), with male counts generally exceeding female counts across the four sampling areas (**Figure 3**). Of the total collection, 183 specimens (55.3%) were morphologically identified as *Phlebotomus* and 146 (44.1%) as *Sergentomyia*, while two damaged male specimens (0.6%) could not be assigned to genus. *Phlebotomus* comprised 121 males and 62 females, whereas *Sergentomyia* comprised 118 males and 28 females (**Figure 4**; **Table 1**). A significant association was observed between genus and sex among the 329 specimens identified to genus level (Pearson’s χ²(1) = 8.83, p = 0.003; Cramer’s V = 0.164).

**Figure 2:**
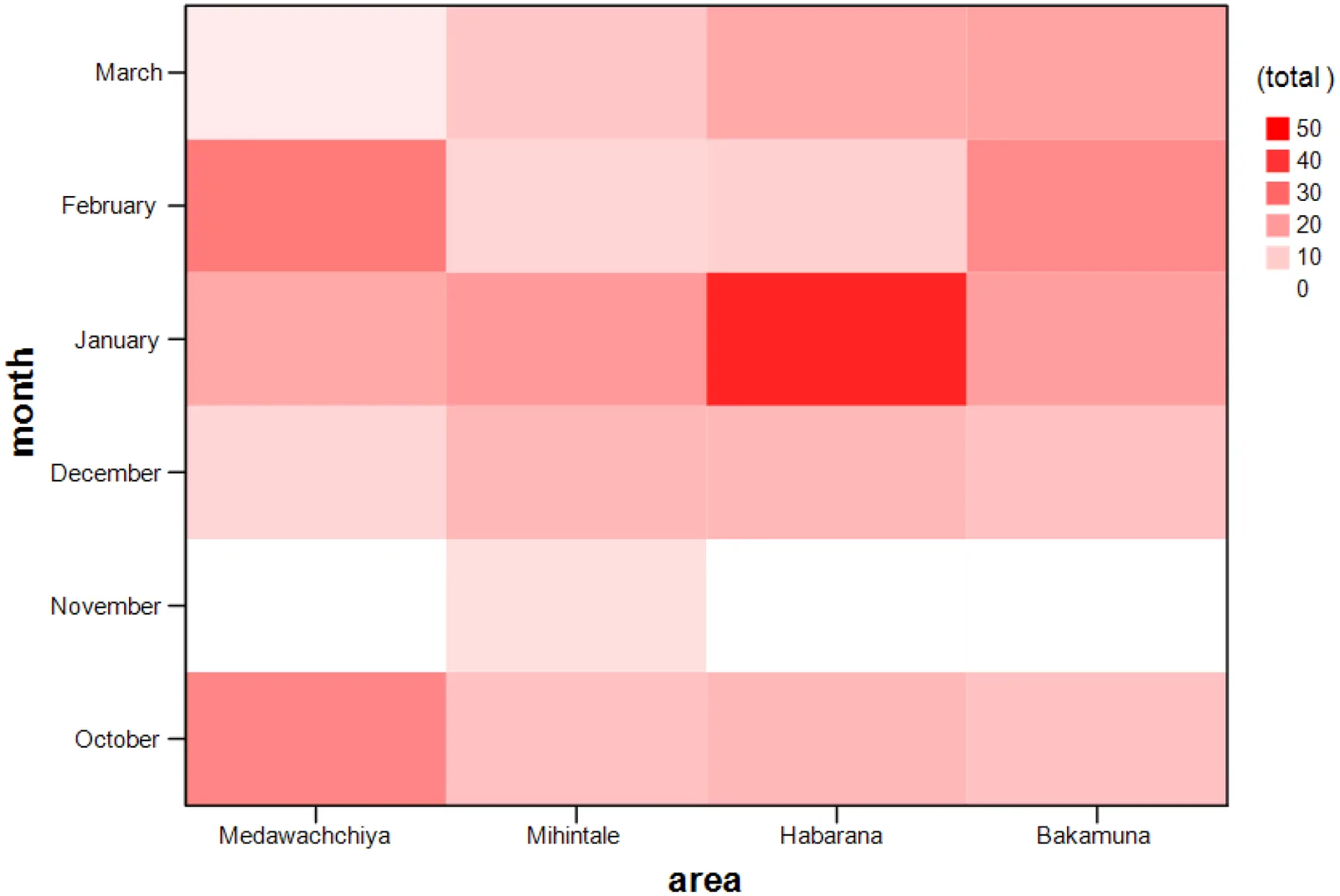
Heatmap showing monthly variation in sand fly abundance across the four sampling areas from October 2024 to March 2025. Each cell represents the total number of sand flies collected at a given site during the corresponding month, with darker red shading indicating greater abundance.

**Figure 3:**
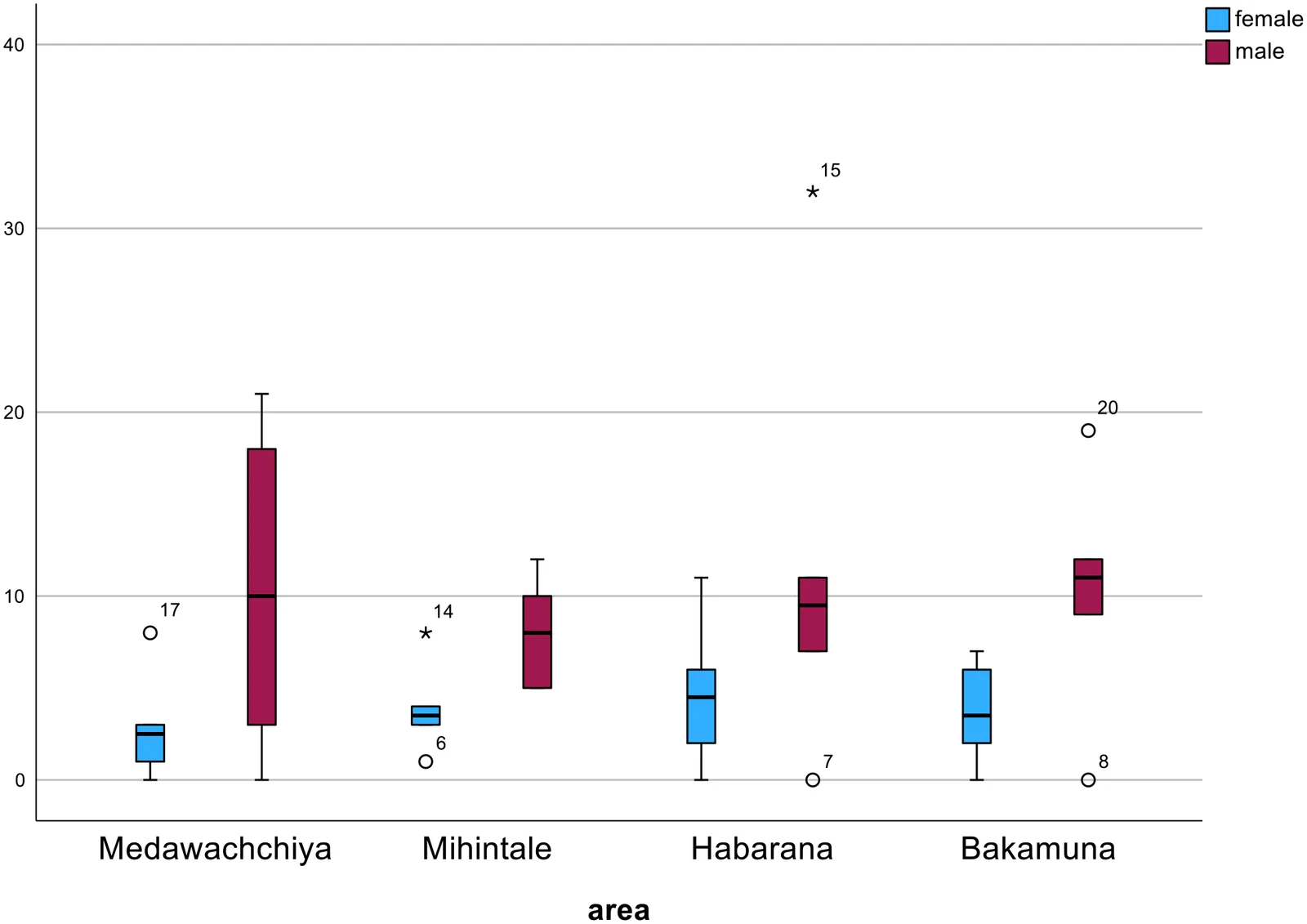
Distribution of monthly female and male sand fly counts across the four sampling areas from October 2024 to March 2025. Boxes represent the interquartile range, horizontal lines indicate medians, and whiskers show the non-outlying range. Circles and asterisks denote outlying and extreme observations, respectively; adjacent numbers are case identifiers.

**Figure 4:**
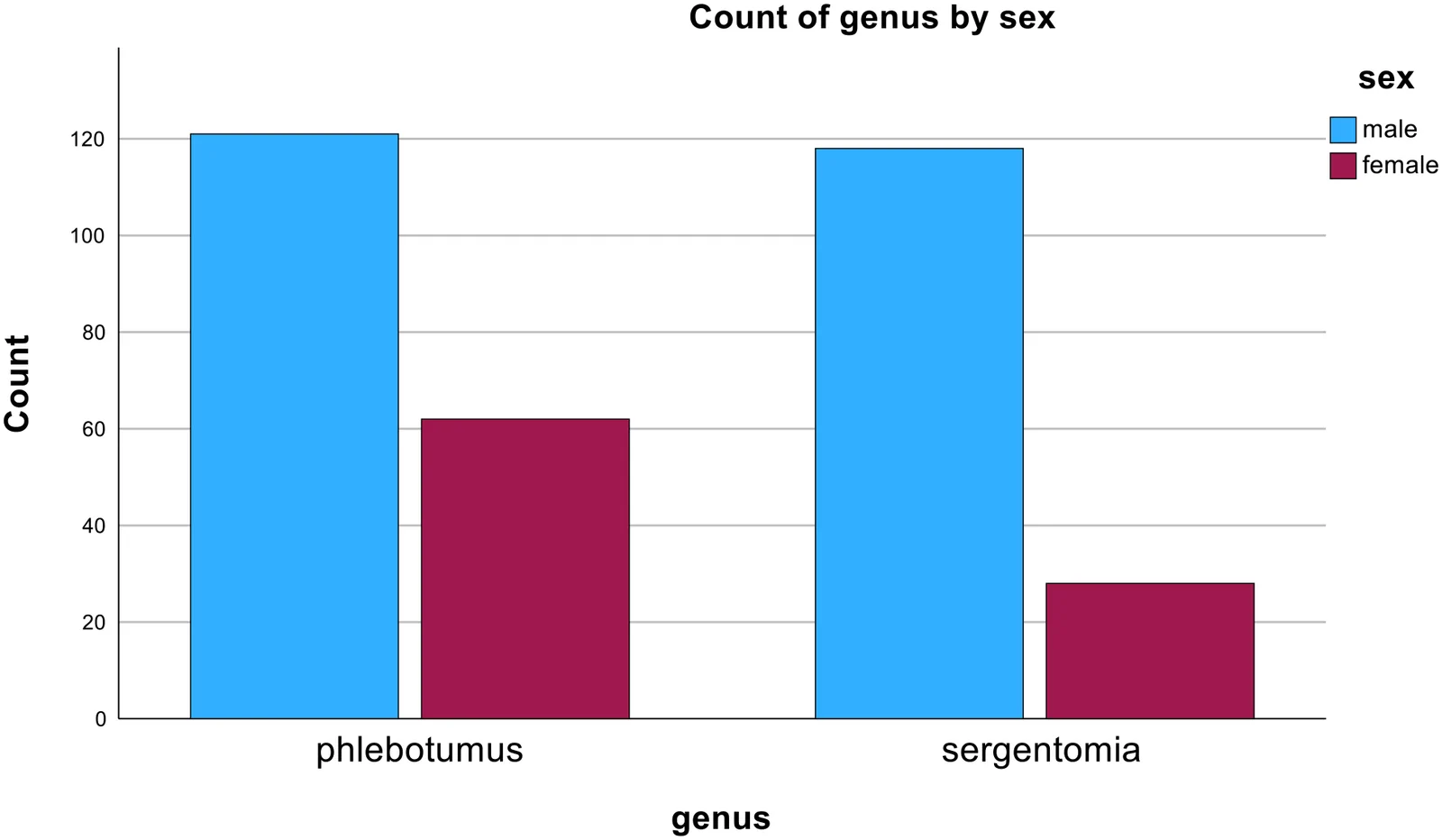
Distribution of morphologically identified sand flies by genus and sex. *Phlebotomus* comprised 121 males and 62 females, whereas *Sergentomyia* comprised 118 males and 28 females.

**Table 1:** Summary of sand fly sample collection details, including the number of male and female specimens obtained.

Genus/category	Male, n (%of total)	Female, n (%of total	Total, n (%of total
*Phlebotomus*	121 (36.6%)	62 (18.7%)	183 (55.3%)
*Sergentomyia*	118 (35.6%)	28 (8.5%)	146 (44.1%)
Damaged	2 (0.6%)	0 (0.0%)	2 (0.6%)
%Overall Total	**241 (72.8%)**	**90 (27.2%)**	**331 (100.0%)**

Bold indicate the overall totals of male, female, and total sand fly specimens collected.

### 3.2 Molecular prevalence of *Wolbachia* endosymbionts

To assess detectable *Wolbachia* infections among the sampled females, molecular screening was conducted on all 90 field-collected female sand flies. Genomic DNA was extracted from 90 female specimens representing four collection sites and a six-month sampling period. PCR amplification targeting the *wsp* gene did not detect *Wolbachia* in any of the 90 samples. Accordingly, *Wolbachia* was not detected among the examined females (0/90; observed prevalence, 0.0%; approximate rule-of-three upper 95% prevalence limit, 3.3%) (**Table 2**). The positive control produced an amplicon of the expected size, whereas the no-template negative control showed no amplification, supporting the expected assay performance and the absence of detectable contamination in the PCR reagents.

**Table 2:** Summary of *Wolbachia* endosymbiont screening in female sand flies collected from the study sites, showing detection outcomes.

Target gene	Genus tested	Total samples tested (N)	Positive samples (n)	Prevalence rate (%)
*wsp*	*Phlebotomus* sp.	62	0	0.0
*wsp*	*Sergentomyia* sp.	28	0	0.0
**Total**	**All Genera**	**90**	**0**	**0.0**

Bold indicate the overall totals across all tested genera, including the total number of samples tested, positive samples, and overall prevalence rate.

### 3.3 16S rRNA gene sequencing and bacterial community composition

A total of 432,078 reads were obtained from the pooled sample. After quality filtering, 241,604 reads (55.92%) were retained, of which 227,572 were denoised and 220,698 (51.08% of input) remained after chimera removal. The final unfiltered feature table contained 883 ASVs. Domain-level classification assigned 113,473 reads (51.42%) to Bacteria, 15,979 (7.24%) to Eukaryota and 3 (0.0014%) to Archaea, while 91,243 reads (41.34%) remained unassigned. After exclusion of 141 mitochondrial reads, the bacterial table contained 113,332 reads and 125 ASVs. *Proteobacteria* accounted for 99.75% of bacterial reads. *Enterobacterales* was the predominant order (89.69%), followed by *Xanthomonadales* (5.28%). Yersiniaceae accounted for 47.10% of bacterial reads, while 37.12% could not be resolved below *Enterobacterales*. *Enterobacteriaceae* and *Xanthomonadaceae* represented 5.47% and 5.28%, respectively. *Serratia* was the most abundant identifiable genus (47,274 reads; 41.71% of bacterial reads and 21.42% of all non-chimeric reads). *Stenotrophomonas* accounted for 5,313 reads (4.69% of bacterial reads; 2.41% of all non-chimeric reads). Other named genera each represented less than 1% of bacterial reads (**Figure 5**). The relative taxonomic composition of the microbial community is shown in **Figure 6**.

**Figure 5:**
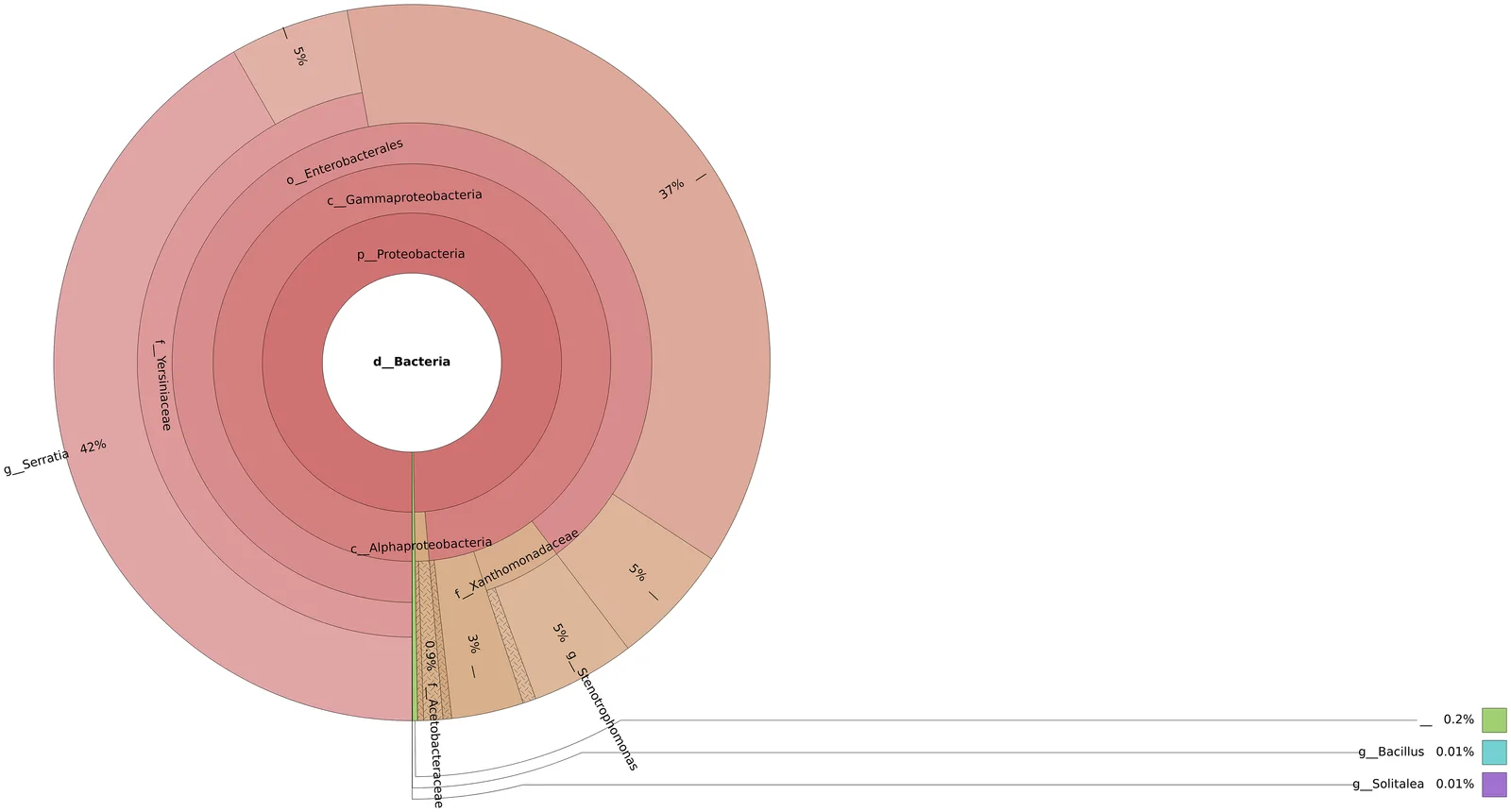
Krona plot illustrating the hierarchical taxonomic distribution of bacterial taxa identified in phlebotomine sand flies.

**Figure 6:**
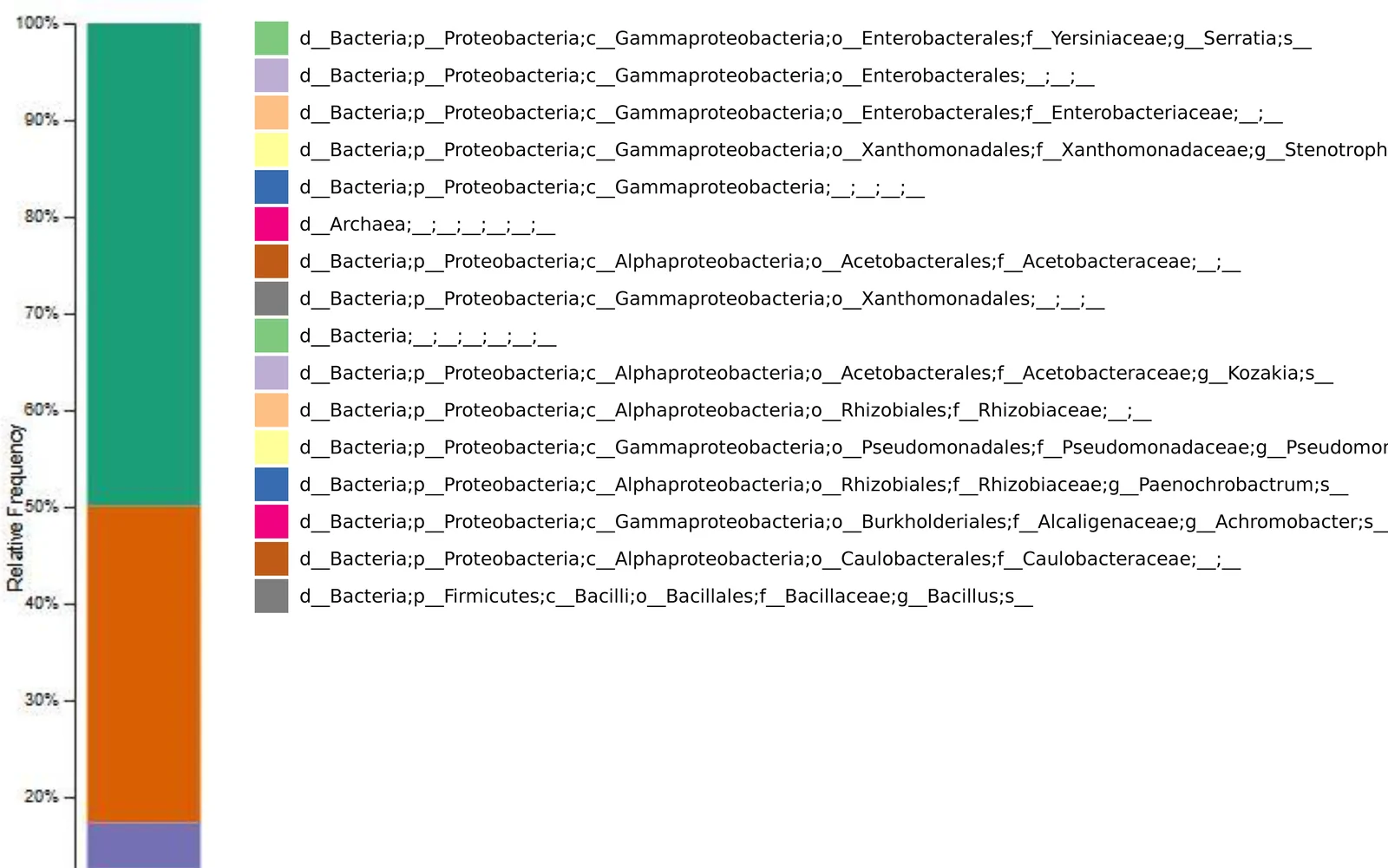
Relative taxonomic composition of the microbial community detected in the pooled sample of 25 female sand flies using 16S rRNA gene amplicon sequencing. The stacked bar represents the relative abundance of taxonomic lineages assigned against the SILVA reference database, with each colour indicating a distinct lineage. Serratia was the most abundant genus-level assignment.

### 3.4 *Wolbachia* screening within the 16S dataset

No ASV was assigned to *Wolbachia* by the SILVA-based Naive Bayes classifier. A preliminary search restricted to an NCBI-derived *Wolbachia* reference collection returned 24 provisional candidate ASVs. However, competitive alignment of these candidates against the complete SILVA 138.1 SSU Ref NR99 database reassigned all candidates to non-*Wolbachia* taxa. Thus, no competitively confirmed *Wolbachia* ASV was detected at thresholds of at least 97% sequence identity and 90% query coverage. The pooled 16S analysis therefore provided complementary evidence that *Wolbachia* was not detected in the analysed pool under the applied bioinformatic criteria.

## 4 Discussion

To our knowledge, this study represents the first study to investigate natural *Wolbachia* endosymbionts in phlebotomine sand fly populations in Sri Lanka using targeted PCR and complementary 16S rRNA gene amplicon sequencing, specifically targeting endemic cutaneous leishmaniasis (CL) hotspots within the selected high endemic areas in North Central Province. Leishmaniasis remains a significant public health challenge in Sri Lanka, where the transmission cycle is primarily driven by *Leishmania donovani* and its proven vector, *Phlebotomus argentipes (*Karunaweera et al., 2021). Given the global interest in utilizing *Wolbachia* for vector-borne disease control—a strategy proven effective against mosquito-borne pathogens (Simões et al., 2011)—understanding its natural prevalence in local sand fly populations is a critical prerequisite for assessing the feasibility of similar symbiont-mediated control programs. In this study, *Wolbachia* was not detected in any of the 90 female sand flies screened individually using *wsp*-targeted PCR. This finding was supported by 16S rRNA gene amplicon sequencing of a pooled subset of 25 females, which generated 220,698 non-chimeric reads representing 883 ASVs but yielded no competitively confirmed *Wolbachia* assignment under the applied taxonomic criteria. Together, these two approaches provide complementary evidence that *Wolbachia* was not detectable in the specimens examined. However, the observed result of 0/90 should not be interpreted as definitive evidence that the bacterium is absent from the wider regional sand fly population. Based on the conventional rule of three, the approximate upper 95% prevalence limit remains 3.3%, meaning that a low-frequency infection could have remained undetected. These concordant results provide complementary evidence that *Wolbachia*was not detectable in the examined *Phlebotomus* and *Sergentomyia* specimens under the applied methods. Nevertheless, they do not exclude low-density infections or strains with sequence variation affecting *wsp*-based detection, particularly because the 16S analysis was conducted on a single pooled subset. These findings contrasts with several published global epidemiological surveys. For instance, extensive studies have reported *Wolbachia* infection rates ranging from 26% to over 80% among wild *Phlebotomus* and Lutzomyia populations in countries such as Turkey, Iran, and Panama (Ono et al., 2001; Azpurua et al., 2010; Bordbar et al., 2014; Karatepe et al., 2018; Torres-Llamas et al., 2025). Marked taxonomic and geographic variation in *Wolbachia* occurrence has been reported in other sand fly populations. In a large survey of 858 specimens from Türkiye, the overall prevalence was 16.67% (Yilmaz and Kasap, 2026); however, 133 of the 143 positive specimens belonged to *Phlebotomus major*. Among the infected taxa, prevalence ranged from 0.83% in *Ph. tobbi* and 3.03% in *Sergentomyia minuta* to 20.59% in *Ph. papatasi* and 62.74% in *Ph. major* s.l. In contrast, an earlier study of 40 *Phlebotomus* specimens from Antalya reported 70% positivity (Karatepe et al., 2018), illustrating the substantial variation that can occur between sampled populations and study designs. Neotropical surveys have also produced heterogeneous findings, including a prevalence of 2.5% in a Brazilian *Lutzomyia* sample (Da Rocha et al., 2018) and detection in only three of the 20 sand fly species examined in Panama (Azpurua et al., 2010). Collectively, these observations suggest that the occurrence of *Wolbachia* in sand flies varies according to host taxon and geographic setting. However, cross-sectional prevalence surveys alone cannot determine whether these infections are stably maintained over time. When considered alongside previous surveys, our findings further illustrate that *Wolbachia* occurrence in sand flies is heterogeneous rather than uniform across species and locations. A recent genome-based study of *Sergentomyia squamirostris* in Japan recovered complete genomes of both *Wolbachia* and *Candidatus tisiphia* from a field-collected female and subsequently documented frequent co-infection at one locality but much lower infection frequencies at another (Itokawa et al., 2025). This finding supports the possibility of host- and locality-specific patterns but does not establish that the infections were recently acquired or transient. It is therefore more appropriate to describe *Wolbachia* as inconsistently detected across sand fly taxa and populations than to classify it as absent from the sand fly core microbiome, which cannot be evaluated using a single pooled sample.

Several non-mutually exclusive explanations could account for the lack of detectable *Wolbachia* in the present samples. The examined populations may be uninfected, or the infection may occur at a prevalence or bacterial density below the detection limits of the methods used. Environmental conditions could also influence infection dynamics, although this possibility was not directly tested. Experimental studies in *Aedes aegypti* have shown that cyclical heat stress, particularly developmental exposure to temperatures reaching approximately 37 °C, can reduce the density and maternal transmission of some *Wolbachia* strains (Ross et al., 2017). However, these effects vary among *Wolbachia* strains and host backgrounds, and the present study did not directly test whether temperatures in the Sri Lankan dry zone affected Wolbachia density or transmission. Temperature-related effects should therefore be considered a hypothesis for future investigation rather than a demonstrated cause of non-detection.

Interactions with other members of the microbiota may also influence *Wolbachia* establishment. Experimental studies in *Anopheles* mosquitoes have shown that the native microbiota can restrict the vertical transmission of an introduced *Wolbachia* infection, while reciprocal interference between *Asaia* and *Wolbachia* has been observed in the reproductive tissues of some mosquito species (Hughes et al., 2014; Rossi et al., 2015). These findings provide a biological basis for investigating symbiont–symbiont interactions, but they cannot be directly extrapolated to sand flies. In the present study, *Serratia* represented 41.71% of bacterial reads in a single pooled sample. Because the analysis was based on pooled whole females and relative-abundance data, it provides no information on tissue localization, absolute bacterial density, or direct interactions with *Wolbachia*. The dominance of *Serratia* is therefore an observation of interest, but it should not be interpreted as evidence of competitive exclusion without targeted tissue-level or experimental investigation.

Methodological limitations should also be considered. Screening with a single conventional PCR marker may fail to detect infections with low bacterial densities or sequence variation at the *wsp* primer-binding regions, as no single PCR protocol detects all known *Wolbachia* lineages with equal sensitivity (Simões et al., 2011). The pooled 16S rRNA gene amplicon dataset provides useful complementary evidence but does not verify the infection status of each PCR-screened individual. Pooling may dilute a rare or low-density infection, while amplification and taxonomic-classification biases may also affect detection. The positive and no-template controls confirm that the PCR reagents and cycling conditions functioned as expected and that detectable contamination was absent; however, they do not independently demonstrate the amplifiability of DNA from every sand fly specimen. Accordingly, the combined findings support an absence of detectable *Wolbachia* under the applied methods, but they do not fully exclude low-density or genetically divergent infections. Future surveillance incorporating an internal host-gene control, additional *Wolbachia* markers such as 16S rRNA and *ftsZ*, quantitative or digital PCR, and broader individual-level sampling across sites and seasons would provide a more sensitive and comprehensive assessment.

Furthermore, a notable limitation of the present study is the taxonomic resolution of the examined specimens. Although *Phlebotomus argentipes* is the principal vector of leishmaniasis in Sri Lanka, the specimens analyzed in the present study were identified only to genus level, therefore cannot be confidently assigned to *P. argentipes*. As highlighted in recent literature, accurate species identification of phlebotomine sand flies is crucial for developing effective preventive and control measures, which, in turn, support the assessment of the risk of local leishmaniasis outbreaks (Soomro et al., 2025). In our study, the 16S rRNA amplicon sequencing was specifically restricted to a pooled sample of *Phlebotomus* females. Since *Phlebotomus* species (particularly *P. argentipes*) are the established vectors of leishmaniasis in Sri Lanka, while *Sergentomyia* species are generally considered non-vectors, this targeted approach allowed us to confidently attribute the observed microbial community—most notably the overwhelming dominance of *Serratia*—directly to the relevant vector genus without the confounding biological background of non-vector species.

Moving beyond the targeted detection of *Wolbachia*, 16S rRNA gene amplicon sequencing provided a descriptive profile of bacterial DNA detected in the single pooled sample of female sand flies. After the exclusion of mitochondrial sequences, the bacterial dataset contained 113,332 reads representing 125 ASVs. Proteobacteria accounted for 99.75% of these reads, and *Serratia* was the most abundant identifiable genus, comprising 47,274 reads, equivalent to 41.71% of bacterial reads and 21.42% of all non-chimeric reads. Although the present study did not directly examine functional interactions between *Serratia* and *Wolbachia*, findings from other dipteran models provide a basis for a testable hypothesis. In *Lutzomyia longipalpis*, predicted metagenomic analyses identified differences in community-level iron- and phosphate-transport pathways between blood-fed and *Leishmania*-infected midguts; however, these functions were not demonstrated to be specific to *Serratia* (Petrosino et al., 2017). Experimental exposure to *S. marcescens* also elicited a reactive oxygen species-mediated immune response in *Lu. longipalpis* (Diaz-Albiter et al., 2012), while *Wolbachia* transinfection of sand fly-derived cell lines altered the expression of genes associated with the IMD, Toll and Jak–STAT pathways (Da Silva Gonçalves et al., 2019). In *Aedes aegypti*, *Serratia* density increased markedly following a blood meal, although comparable proliferation has not yet been demonstrated in sand flies (Kozlova et al., 2021). Some *S. marcescens* strains have also shown lytic activity against *Leishmania* promastigotes, but this effect appears to be strain- and mechanism-dependent, and prodigiosin was not identified as the principal lytic factor in one experimental model (Moraes et al., 2009). Furthermore, studies in mosquitoes have shown that the native microbiota, including *Asaia*, can interfere with *Wolbachia* colonization and maternal transmission (Hughes et al., 2014; Rossi et al., 2015). Collectively, these studies demonstrate that resident bacteria can be associated with changes in microbial community functions and host responses in other dipteran models. However, they do not demonstrate that Serratia influences Wolbachia establishment in sand flies. The pooled relative-abundance data generated in the present study cannot test direct microbial interactions or competitive exclusion. Therefore, any potential interaction between Serratia and Wolbachia should be regarded only as a working hypothesis requiring validation through individual-level absolute quantification, tissue-specific localization, and controlled co-colonization experiments.

The epidemiology of *Leishmania donovani* in the Indian subcontinent typically presents as Visceral Leishmaniasis (VL) (Chowdhury et al., 2016). However, in the Sri Lankan region, an endemic strain of *L. donovani* predominantly causes Cutaneous Leishmaniasis (CL), which is characterized mainly by localized skin lesions (Beeching et al., 2007). Diaz-Albiter et al. (2012) demonstrated that oral exposure to *S. marcescens* increased reactive oxygen species production in the midgut of *Lutzomyia longipalpis* and that experimentally increased oxidative activity reduced the survival of *Leishmania mexicana* (Diaz-Albiter et al., 2012). However, these observations from an experimental model cannot be extrapolated directly to Sri Lankan sand flies. The present study did not measure oxidative activity, parasite burden, or vector–parasite interactions; therefore, the current data do not support an association between Serratia and either *L. donovani* development in sand flies or the predominantly cutaneous presentation of infection in Sri Lanka. Any such association would require investigation through controlled vector–microbiome–parasite studies. A substantial proportion of the non-chimeric reads remained unassigned at the domain level (91,243 reads; 41.34%), while a further 15,979 reads (7.24%) were classified as *Eukaryota*. This unresolved fraction may reflect the combined effects of the mixed hypervariable-region library design, variation in read length, the potential presence of residual proprietary primer sequences and the presence of non-target DNA in the pooled whole-insect sample (Barb et al., 2016). Non-specific amplification of host-derived mitochondrial or other eukaryotic rRNA sequences has previously been observed in insect microbiome studies using broadly conserved 16S primers (Novakova et al., 2017). However, because the unassigned and eukaryotic reads were not subjected to a validated host-specific classification procedure in the present study, their biological origin cannot be determined.

In summary, *Wolbachia* was not detected among the 90 individually screened female sand flies (0/90; approximate rule-of-three upper 95% prevalence limit, 3.3%) or in the pooled 16S rRNA gene amplicon dataset. These findings should be interpreted as an absence of detectable infection within the examined samples under the methods applied, rather than as confirmation of its complete absence from sand fly populations in the North Central Province. Nevertheless, the study provides useful baseline evidence from Sri Lanka and is consistent with previous observations that natural sand fly–*Wolbachia* associations can vary considerably among host taxa, populations and geographical locations. Larger, multi-site and multi-season investigations incorporating species-level host identification, multiple *Wolbachia* markers, absolute quantification and complementary sequencing approaches are required to determine whether low-density or geographically restricted infections occur. Controlled laboratory studies are also needed to establish whether *Wolbachia* can be artificially introduced into sand flies, maintained across generations and localized within reproductive tissues, as well as whether such infections affect host fitness, *Leishmania* development or vector competence. The broader bacterial profile identified in the present pooled sample also supports further investigation of sand fly-associated microorganisms as potential candidates for microbiome-informed or paratransgenic control. However, candidate bacteria would require isolate-level characterization and experimental assessment of their safety, colonization stability, transmission and anti-*Leishmania* activity before their suitability for vector-control applications could be determined.

## Data Availability

The datasets presented in this study can be found in online repositories. The names of the repository/repositories and accession number(s) can be found in the article/supplementary material.
