# Supplementary material for: Molecular screening for Wolbachia endosymbionts in phlebotomine sand flies within the North Central Province, Sri Lanka
Source: Front Microbiomes. 2026 Oct 1;5:1905698. doi: 10.3389/frmbi.2026.1905698 (PMC13670030; doi:10.3389/frmbi.2026.1905698)
Supplement: Supplementary file 1 [file Supplementaryfile1.docx]

Supplementary Material

# 1 Supplementary Data

**Blast results of unclassified sequences.**

# 2 Supplementary Figures and Tables

## 2.1 Supplementary Figures2.2. Documented gel images of PCR

13	12	11	10	9	8	7	6	5	4	3	2	1	-	+	L

26	25	24	23	22	21	20	19	18	17	16	15	14	-	+	L

39	38	37	36	35	34	33	32	31	30	29	28	27	-	+	L

52	51	50	49	48	47	46	45	44	43	42	41	40	-	+	L

65	64	63	62	61	60	59	58	57	56	55	54	53	-	+	L

78	77	76	75	74	73	72	71	70	69	68	67	66	-	+	L

90	89	88	87	86	85	84	83	82	81	80	79	+	L	-	B
